# Supplementary figures and images for: Syngeneic model of carcinogen-induced tumor mimics basal/squamous, stromal-rich, and neuroendocrine molecular and immunological features of muscle-invasive bladder cancer
Source: Front Oncol. 2023 Feb 3;13:1120329. doi: 10.3389/fonc.2023.1120329 (PMC9936245; doi:10.3389/fonc.2023.1120329)

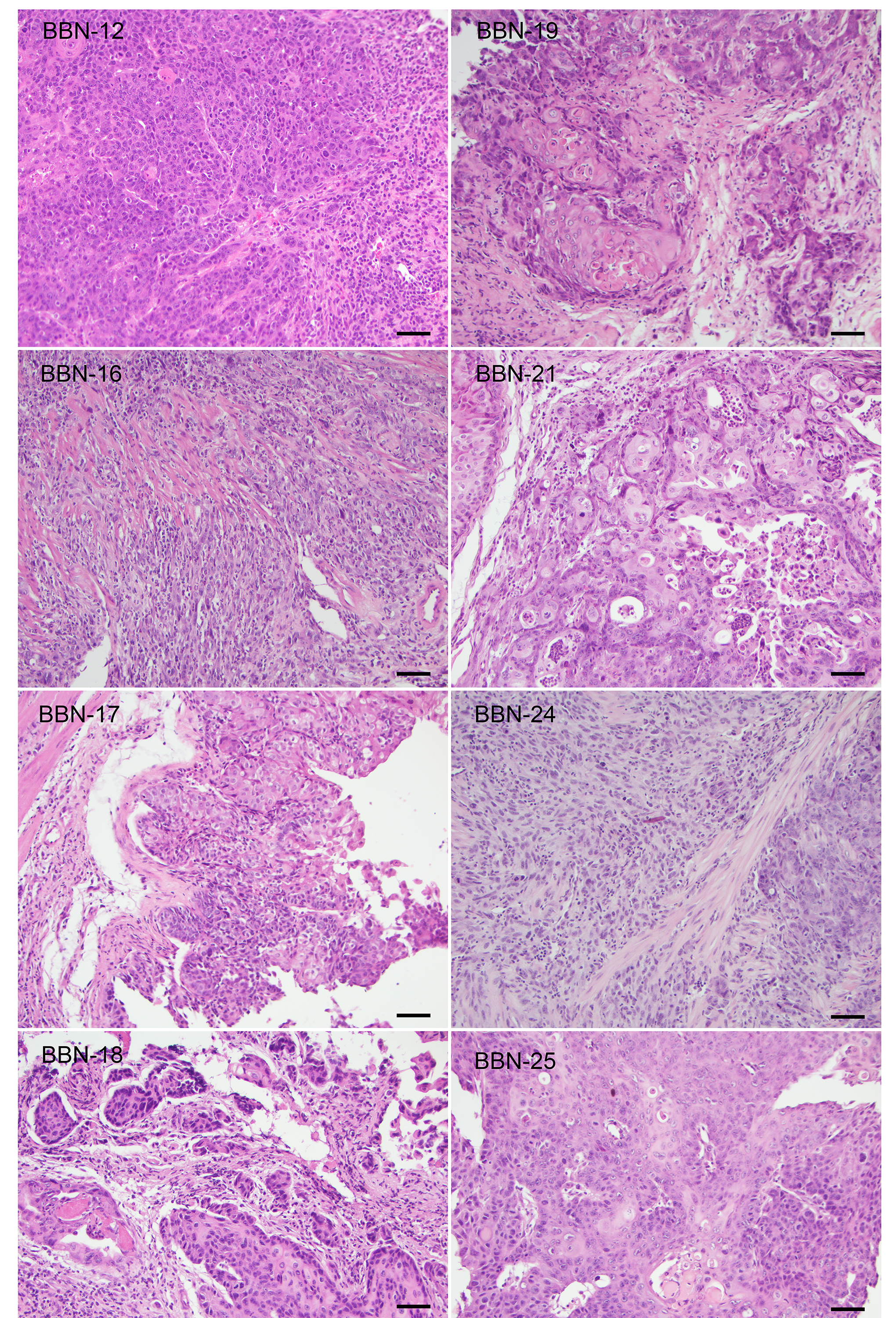

Supplement: Supplementary Figure 1 — High magnification images of original high-grade muscle-invasive tumors (BBN Donor Tumor), stained with H&E. Representative H&E from each original BBN Donor Tumor. Scale bars equal 50µm. [file Image_1.tif]

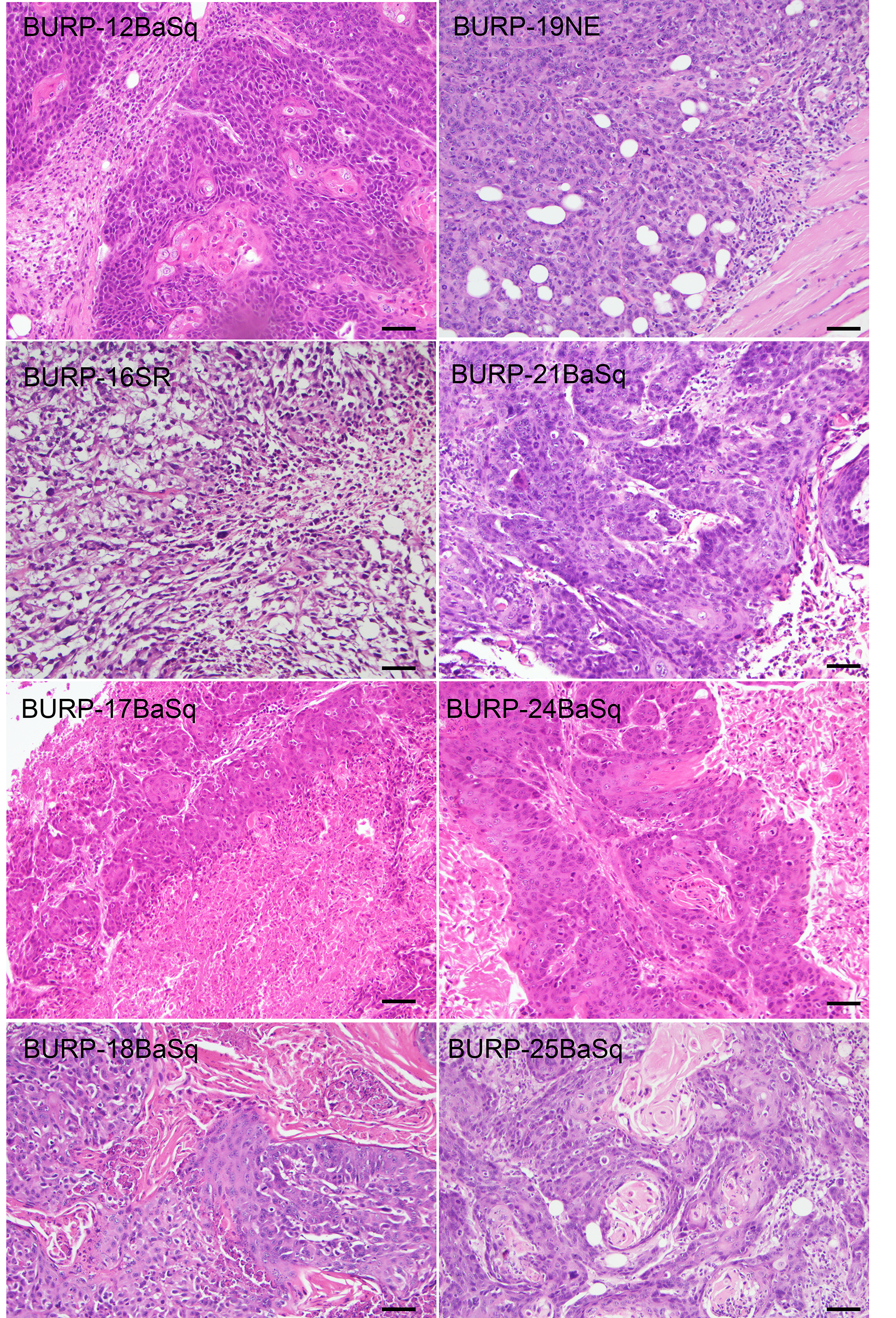

Supplement: Supplementary Figure 2 — High magnification images of the BURP tumor lines. Representative H&E from each established BURP tumor lines. Scale bars equal 50µm. [file Image_2.tif]

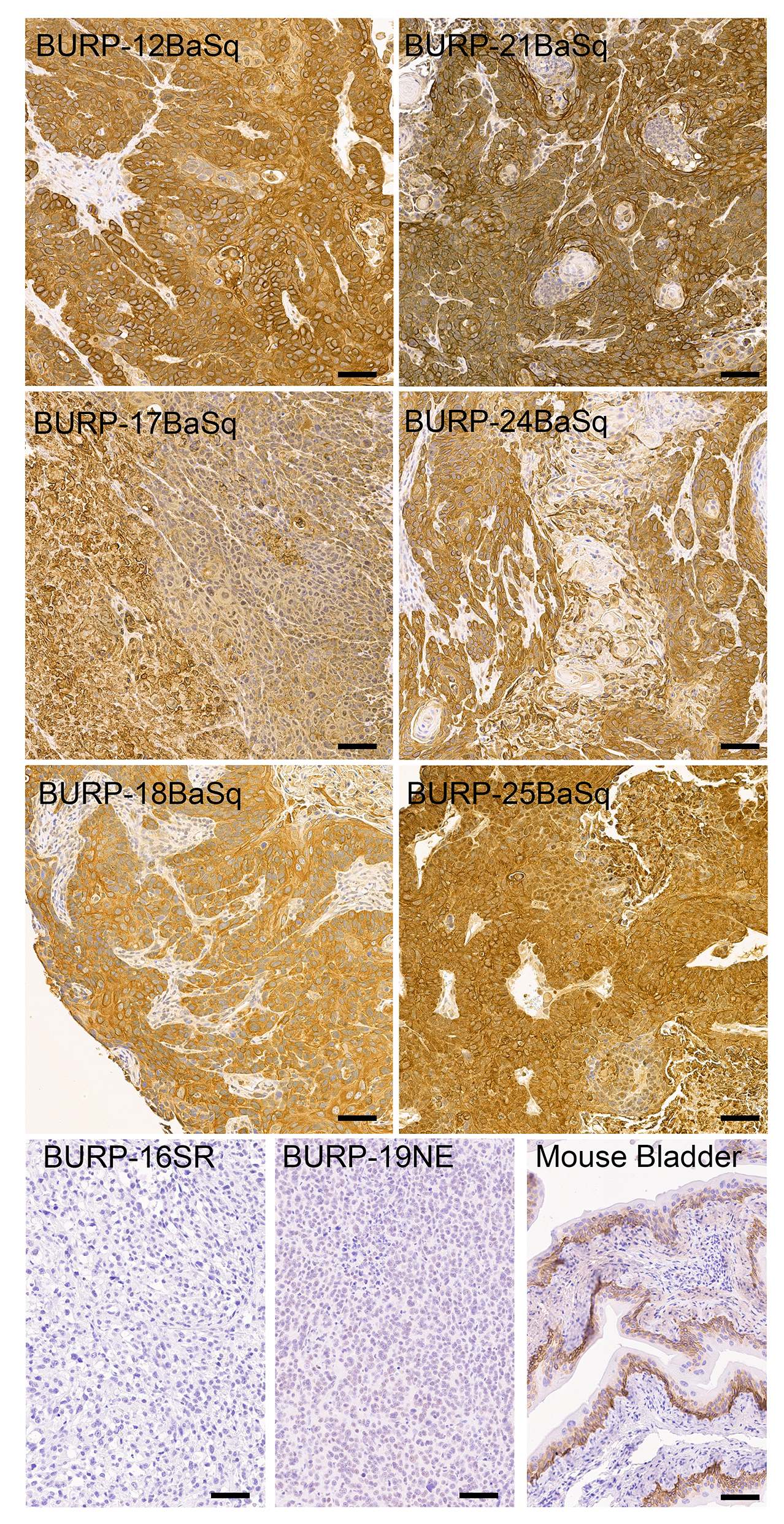

Supplement: Supplementary Figure 3 — IHC of cytokeratin 5 in the BURP lines. BURP-12, -17, -18, -21, -24, -25BaSq express high levels of cytokeratin 5. BURP-16SR and BURP-19NE do not express cytokeratin 5. Cytokeratin 5 is expressed by the basal layer in the mouse bladder (control). Scale bars equal 50µm. [file Image_3.tif]

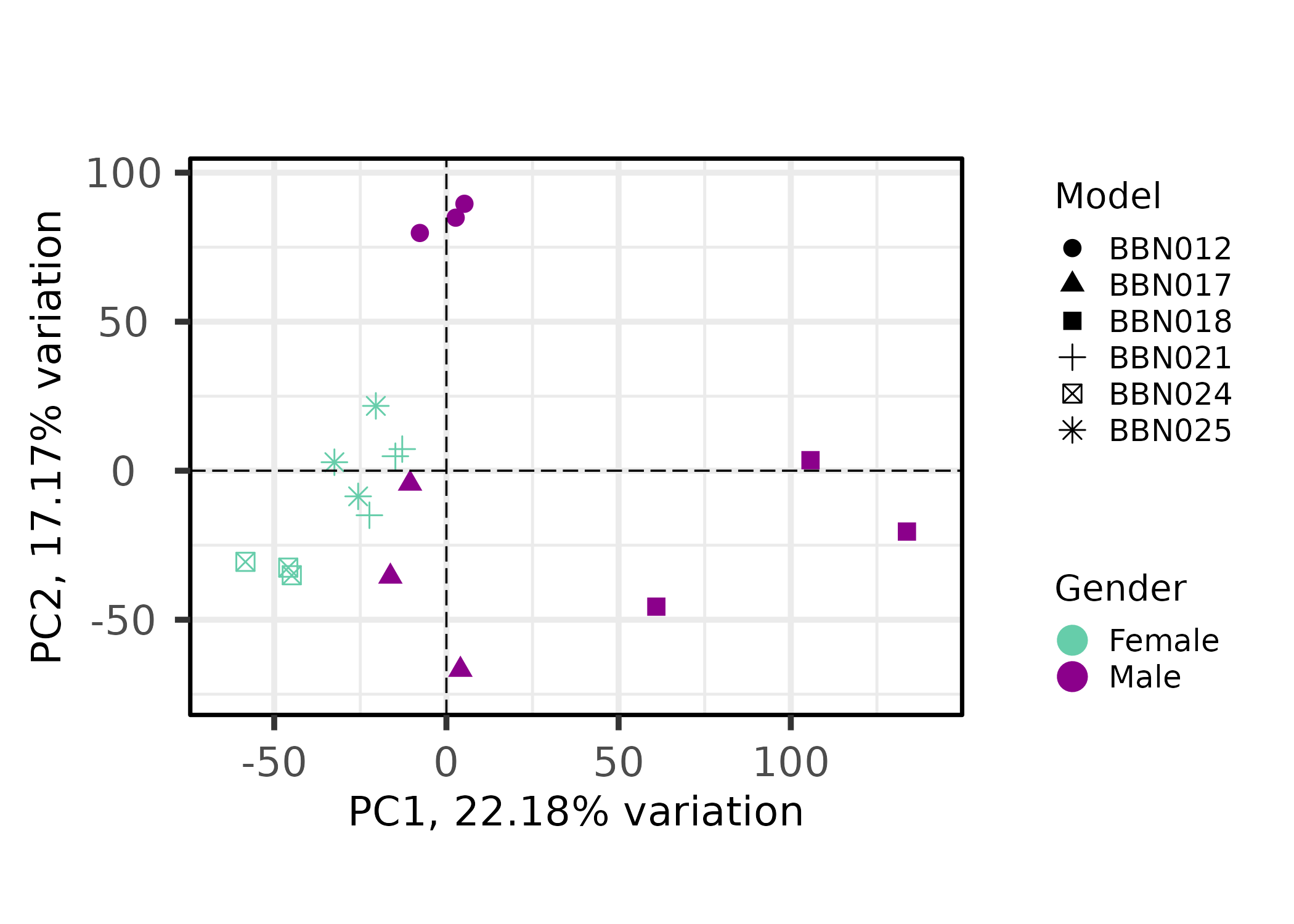

Supplement: Supplementary Figure 4 — Comparison of male and female Ba/Sq BURP tumor lines. PCA plot derived from total transcriptomes of Ba/Sq BURP tumor lines. Each data point represents an individual tumor, with the shape representing the BURP tumor line and the color representing the sex of mouse from which the tumor line was derived. Top two PCs explaining majority of variation are shown. [file Image_4.tiff]

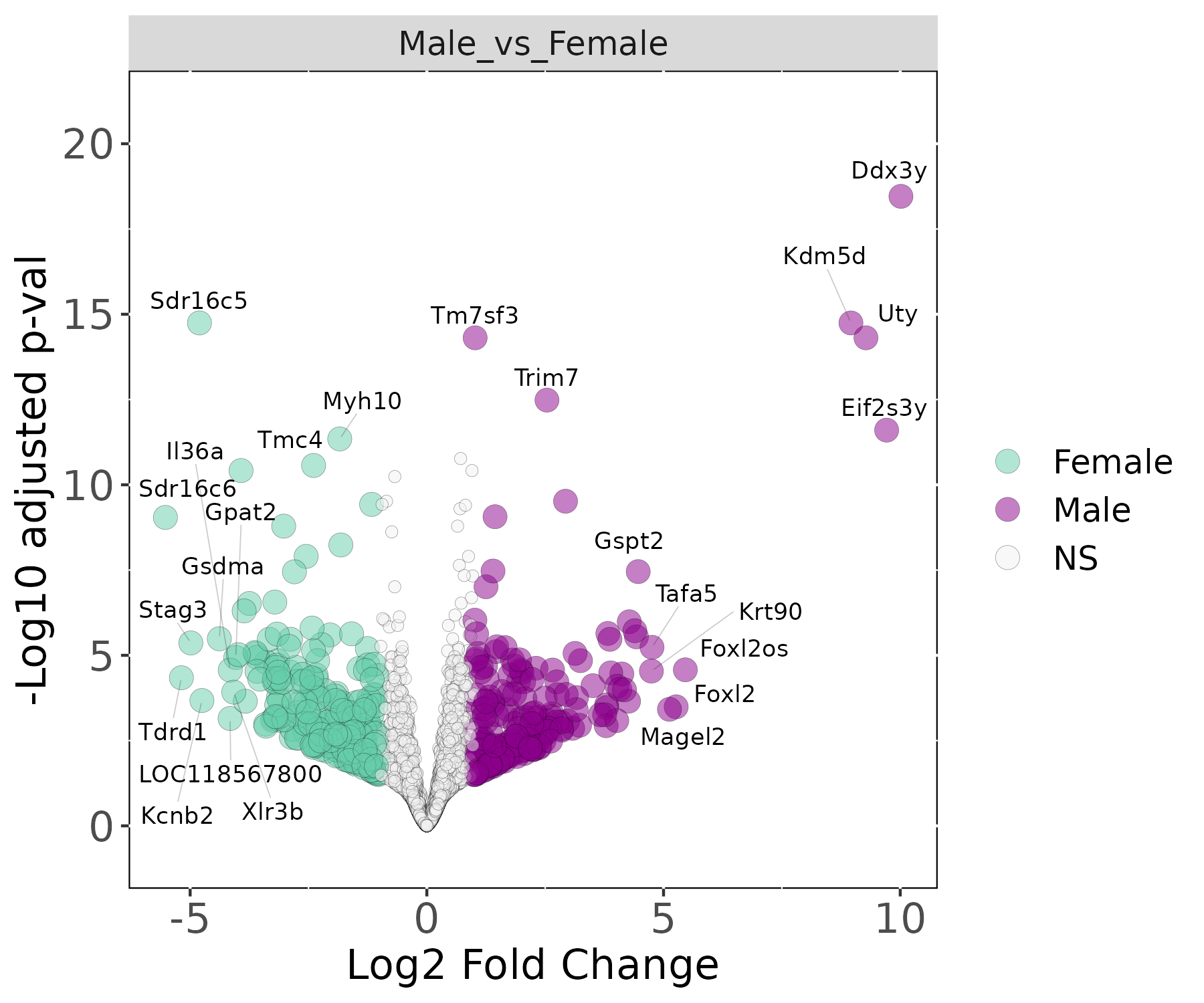

Supplement: Supplementary Figure 5 — Volcano plot depicting differential expression analysis. Highlighted genes represent significant (p.adj < 0.05, log2FC > +/-1) DEGs. [file Image_5.tiff]

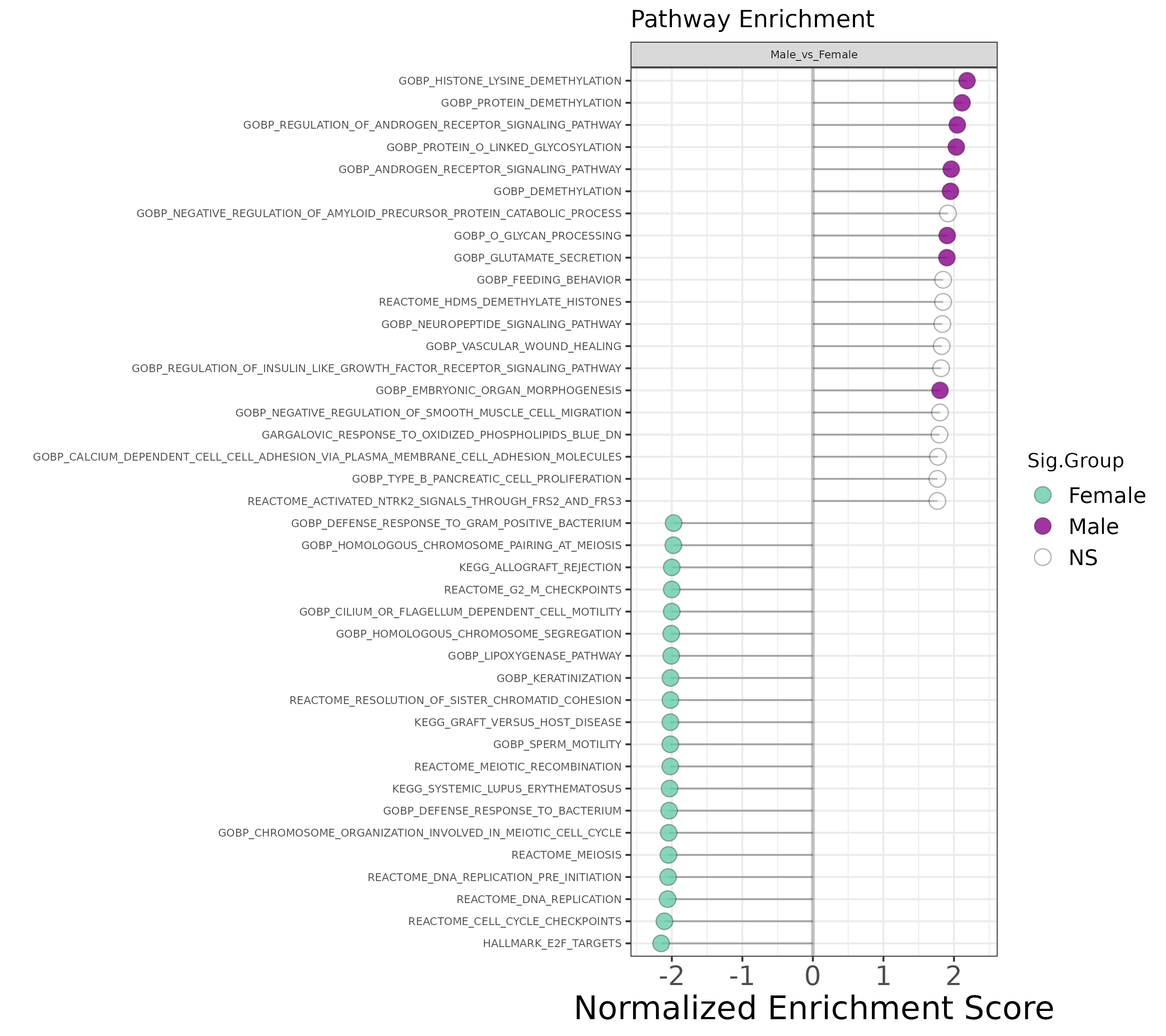

Supplement: Supplementary Figure 6 — Top enriched pathways in male and female transcriptomes via GSEA. Highlighted pathways represent significant enrichments (p.adj < 0.05). [file Image_6.tiff]
