# Supplementary material for: Syngeneic model of carcinogen-induced tumor mimics basal/squamous, stromal-rich, and neuroendocrine molecular and immunological features of muscle-invasive bladder cancer
Source: Front Oncol. 2023 Feb 3;13:1120329. doi: 10.3389/fonc.2023.1120329 (PMC9936245; doi:10.3389/fonc.2023.1120329)
Supplement: Supplementary Table 1 — Gene list of differential expression analysis of male and female Ba/Sq BURP tumor lines. Table representing all significant (p.adj < 0.05, log2FC > +/-1) DEGs derived from comparison of male and female Ba/Sq BURP tumor lines. Genes are ranked by log2FC from the most downregulated in male/female to the most upregulated in male/female. [file Table_1.docx]

| **Gene** | **baseMean** | **log2FoldChange** | **lfcSE** | **pvalue** | **padj** |
| --- | --- | --- | --- | --- | --- |
| **Sdr16c6** | 22.42145965 | -5.5223655 | 0.944958 | 1.31E-12 | 9.07E-10 |
| **Tdrd1** | 31.69185506 | -5.1850957 | 1.569972 | 5.07E-07 | 4.55E-05 |
| **Stag3** | 260.1727137 | -4.985421 | 1.152984 | 2.00E-08 | 4.34E-06 |
| **Sdr16c5** | 55.89351389 | -4.803392 | 0.579365 | 3.71E-19 | 1.80E-15 |
| **Kcnb2** | 16.71898105 | -4.7510549 | 1.81265 | 3.85E-06 | 0.000211 |
| **Gsdma** | 68.08687429 | -4.3834083 | 0.914513 | 1.42E-08 | 3.27E-06 |
| **LOC118567800** | 12.33072074 | -4.1564541 | 2.213694 | 1.94E-05 | 0.000712 |
| **Il36a** | 24.80585587 | -4.1536134 | 0.984278 | 2.52E-07 | 2.72E-05 |
| **Xlr3b** | 73.27402316 | -4.0785091 | 1.145423 | 1.79E-06 | 0.00012 |
| **Gpat2** | 22.30406194 | -4.0357496 | 0.873171 | 7.48E-08 | 1.16E-05 |
| **Lce3b** | 59.07185057 | -3.9820613 | 0.844612 | 5.79E-08 | 9.55E-06 |
| **Cfap57** | 27.72946393 | -3.9250363 | 0.545735 | 3.09E-14 | 3.83E-11 |
| **Susd4** | 25.83187778 | -3.859035 | 0.696521 | 1.37E-09 | 4.98E-07 |
| **Pla2g4d** | 27.16572084 | -3.8350819 | 1.144723 | 4.09E-06 | 0.000222 |
| **Slc26a9** | 122.7745743 | -3.7497829 | 0.660397 | 8.12E-10 | 3.02E-07 |
| **Greb1** | 112.9647484 | -3.6293385 | 0.73645 | 4.75E-08 | 8.31E-06 |
| **Cpa4** | 102.4923247 | -3.6098811 | 0.734489 | 5.21E-08 | 8.81E-06 |
| **Lce3e** | 72.1371862 | -3.5870952 | 0.796874 | 2.87E-07 | 2.96E-05 |
| **Dlx3** | 56.73500415 | -3.5820903 | 0.738747 | 7.30E-08 | 1.14E-05 |
| **Them5** | 11.15885602 | -3.5274802 | 0.81641 | 5.80E-07 | 4.99E-05 |
| **Pcdha8** | 11.16957368 | -3.4051864 | 1.487648 | 3.44E-05 | 0.001048 |
| **Ppp2r2c** | 70.19276553 | -3.3985668 | 1.603249 | 4.11E-05 | 0.001197 |
| **Cdsn** | 677.601247 | -3.3911059 | 0.706595 | 1.20E-07 | 1.60E-05 |
| **Kprp** | 156.9981975 | -3.3283386 | 0.631303 | 1.49E-08 | 3.38E-06 |
| **H19** | 2972.691202 | -3.2587458 | 1.186629 | 2.30E-05 | 0.000804 |
| **Chrna5** | 4.807829643 | -3.2387513 | 1.130785 | 1.97E-05 | 0.000719 |
| **Endou** | 369.5455048 | -3.2256913 | 0.740114 | 7.26E-07 | 5.82E-05 |
| **Psors1c2** | 36.01538525 | -3.2156053 | 0.723538 | 5.52E-07 | 4.83E-05 |
| **Chst5** | 8.366951365 | -3.2116284 | 0.547845 | 7.00E-10 | 2.75E-07 |
| **Tmem266** | 32.99036435 | -3.1957057 | 1.060692 | 1.62E-05 | 0.000628 |
| **Pnma2** | 26.32300171 | -3.1947783 | 0.738384 | 8.24E-07 | 6.33E-05 |
| **Gpa33** | 149.3875014 | -3.191688 | 0.672073 | 1.85E-07 | 2.26E-05 |
| **Pnpla1** | 49.61600737 | -3.1711248 | 0.88516 | 5.70E-06 | 0.000283 |
| **Misp** | 122.3768982 | -3.1696219 | 1.067139 | 1.78E-05 | 0.000666 |
| **Efcab1** | 16.70538623 | -3.1681269 | 0.660507 | 1.56E-07 | 1.96E-05 |
| **Nrn1** | 38.12039366 | -3.1617418 | 0.585044 | 9.36E-09 | 2.39E-06 |
| **Cap2** | 17.64129541 | -3.1508438 | 0.641748 | 9.91E-08 | 1.43E-05 |
| **Tlcd4** | 20.26067434 | -3.142044 | 0.679238 | 3.10E-07 | 3.11E-05 |
| **Lce1a1** | 16.44769607 | -3.1365045 | 0.810392 | 3.04E-06 | 0.000175 |
| **Sdr9c7** | 175.7098658 | -3.1279594 | 0.699588 | 5.43E-07 | 4.79E-05 |
| **Lce3f** | 62.69563996 | -3.1078877 | 0.821502 | 3.82E-06 | 0.000211 |
| **Lce3d** | 27.76981521 | -3.1058717 | 1.010972 | 1.59E-05 | 0.00062 |
| **Aox4** | 8.028111877 | -3.0973458 | 1.090433 | 2.34E-05 | 0.00081 |
| **Scnn1b** | 29.89419184 | -3.0525383 | 0.697391 | 7.79E-07 | 6.15E-05 |
| **Aldh3b2** | 158.9480309 | -3.0241743 | 0.445837 | 2.51E-12 | 1.63E-09 |
| **Rbm24** | 23.62385291 | -3.0176703 | 1.030898 | 2.17E-05 | 0.000778 |
| **Rorc** | 16.09320666 | -2.913186 | 0.555458 | 2.62E-08 | 5.45E-06 |
| **Gbp10** | 56.54116437 | -2.8848342 | 0.617141 | 3.03E-07 | 3.09E-05 |
| **Alox12b** | 314.6004302 | -2.8799247 | 0.537247 | 1.51E-08 | 3.38E-06 |
| **Col26a1** | 14.40958255 | -2.8625622 | 1.547764 | 0.000109 | 0.002519 |
| **Ggt1** | 11.76770836 | -2.8443073 | 1.162509 | 5.31E-05 | 0.001466 |
| **Rnf222** | 65.12578609 | -2.8053598 | 0.744865 | 4.52E-06 | 0.000235 |
| **Zfp991** | 174.1018329 | -2.7976181 | 0.444036 | 7.59E-11 | 3.56E-08 |
| **Gsta2** | 43.82432575 | -2.7651214 | 0.575418 | 1.90E-07 | 2.26E-05 |
| **Lce1d** | 14.61929821 | -2.7544471 | 1.428846 | 0.000115 | 0.00263 |
| **Gtsf1l** | 19.44402497 | -2.7421609 | 0.862918 | 1.60E-05 | 0.000623 |
| **Gulp1** | 62.9656032 | -2.736061 | 0.617414 | 7.22E-07 | 5.82E-05 |
| **Lce1a2** | 5.616508253 | -2.7253412 | 1.416981 | 0.00012 | 0.002715 |
| **LOC118567674** | 12.92601338 | -2.711296 | 0.804035 | 1.09E-05 | 0.000469 |
| **Apol10b** | 141.743033 | -2.6684462 | 0.707692 | 4.51E-06 | 0.000235 |
| **Insrr** | 18.49958448 | -2.6276875 | 0.569023 | 3.80E-07 | 3.61E-05 |
| **Vsig10l** | 661.4313297 | -2.6089603 | 0.588846 | 7.11E-07 | 5.77E-05 |
| **Cnga3** | 10.62321505 | -2.5796051 | 0.840564 | 2.03E-05 | 0.000732 |
| **Dnah11** | 23.12150654 | -2.5789151 | 1.063699 | 6.54E-05 | 0.001737 |
| **Ptk6** | 50.94528552 | -2.5702374 | 0.911618 | 3.27E-05 | 0.001014 |
| **Pla2g4f** | 79.92849239 | -2.559629 | 0.681893 | 4.60E-06 | 0.000238 |
| **Tmem108** | 28.94336312 | -2.5490012 | 0.391542 | 2.33E-11 | 1.26E-08 |
| **Trex2** | 151.7577064 | -2.5104052 | 0.741706 | 1.05E-05 | 0.000456 |
| **Sh3gl2** | 40.78781013 | -2.4668989 | 0.870544 | 3.22E-05 | 0.001008 |
| **Aloxe3** | 277.0314814 | -2.4362366 | 0.547503 | 6.12E-07 | 5.11E-05 |
| **Zfp992** | 142.7838529 | -2.4330958 | 0.540551 | 5.16E-07 | 4.60E-05 |
| **Crct1** | 510.0326829 | -2.4313099 | 0.867834 | 3.42E-05 | 0.001048 |
| **Il22ra1** | 63.36061972 | -2.4244095 | 0.435056 | 5.67E-09 | 1.58E-06 |
| **Tgm3** | 146.8056971 | -2.4213445 | 1.461471 | 0.000227 | 0.004274 |
| **Lipm** | 217.4443106 | -2.4209174 | 0.572738 | 1.21E-06 | 8.68E-05 |
| **Bpifc** | 224.7278051 | -2.4189979 | 0.579165 | 1.40E-06 | 9.90E-05 |
| **Adgrf2** | 195.1385693 | -2.4108655 | 0.605858 | 2.40E-06 | 0.000148 |
| **Smim22** | 19.06629292 | -2.4032145 | 1.780819 | 0.000279 | 0.004931 |
| **Krt78** | 438.73914 | -2.3931873 | 0.463936 | 4.00E-08 | 7.35E-06 |
| **Tmc4** | 586.4515862 | -2.3926223 | 0.317674 | 1.87E-14 | 2.71E-11 |
| **Muc15** | 118.0592984 | -2.3824355 | 0.728069 | 1.29E-05 | 0.000528 |
| **Adamts18** | 73.85181018 | -2.3808164 | 1.445201 | 0.000241 | 0.004443 |
| **Sp8** | 38.4319795 | -2.3699941 | 1.396426 | 0.00023 | 0.004297 |
| **Gbp4** | 1258.149157 | -2.3409553 | 0.514538 | 4.03E-07 | 3.77E-05 |
| **Il36rn** | 44.95739683 | -2.3390389 | 1.292008 | 0.000203 | 0.003946 |
| **Fut1** | 29.2162283 | -2.3302486 | 0.525832 | 5.89E-07 | 5.03E-05 |
| **Lars2** | 947.077318 | -2.313362 | 0.8041 | 2.89E-05 | 0.000943 |
| **Gucy2c** | 16.5299827 | -2.3087871 | 1.060091 | 0.000112 | 0.002564 |
| **Gbp6** | 901.4493709 | -2.3067983 | 0.468134 | 9.65E-08 | 1.43E-05 |
| **Scnn1g** | 35.55446725 | -2.3013794 | 0.890212 | 5.18E-05 | 0.001435 |
| **Nkpd1** | 157.8067334 | -2.2991175 | 0.598138 | 3.13E-06 | 0.000178 |
| **Eif1ad7** | 35.01252214 | -2.2804045 | 1.32801 | 0.000244 | 0.004488 |
| **Flg** | 6576.994573 | -2.2774058 | 0.588248 | 2.84E-06 | 0.000166 |
| **Cdhr1** | 106.0433559 | -2.2541163 | 0.918537 | 6.68E-05 | 0.001763 |
| **Unc93a** | 17.39605751 | -2.2455122 | 1.11357 | 0.000153 | 0.003238 |
| **Stmn2** | 74.47402774 | -2.2289369 | 0.949536 | 8.23E-05 | 0.002064 |
| **Mpz** | 5.99833972 | -2.2288122 | 1.506113 | 0.000342 | 0.005753 |
| **Frmpd1** | 25.09469273 | -2.2226391 | 0.423722 | 2.27E-08 | 4.84E-06 |
| **Pglyrp3** | 32.30944142 | -2.1760286 | 0.951715 | 9.28E-05 | 0.002249 |
| **Eif1ad3** | 7.291314149 | -2.1668106 | 1.086587 | 0.000164 | 0.00337 |
| **Gbp8** | 175.0308926 | -2.1495457 | 0.534705 | 1.60E-06 | 0.000109 |
| **Cxcl9** | 1714.829667 | -2.1327532 | 0.659576 | 1.18E-05 | 0.000497 |
| **Lncenc1** | 1122.569822 | -2.117294 | 1.447846 | 0.000399 | 0.006392 |
| **Slc5a1** | 203.2123151 | -2.1099692 | 1.063635 | 0.00017 | 0.003439 |
| **Rnf223** | 121.1956902 | -2.1001382 | 0.737436 | 2.76E-05 | 0.000911 |
| **Slurp2** | 5.367776434 | -2.0495848 | 1.14441 | 0.000251 | 0.004583 |
| **Igf2bp3** | 199.5811024 | -2.0453269 | 0.76054 | 3.79E-05 | 0.001124 |
| **Bnipl** | 145.955011 | -2.0436806 | 0.382147 | 1.01E-08 | 2.52E-06 |
| **Ankk1** | 207.3309089 | -2.0426885 | 1.005717 | 0.000156 | 0.003266 |
| **Prlr** | 19.51814893 | -2.0426627 | 1.010525 | 0.000159 | 0.003313 |
| **Khdrbs2** | 177.9158415 | -2.0250168 | 0.698666 | 2.31E-05 | 0.000804 |
| **Abca12** | 873.5176915 | -2.0209989 | 0.836796 | 6.86E-05 | 0.001798 |
| **Tafa2** | 17.3660982 | -2.0204251 | 0.980952 | 0.000147 | 0.003144 |
| **Myom3** | 9.062627244 | -1.960889 | 0.872897 | 9.74E-05 | 0.002334 |
| **Atp6v1c2** | 55.77508143 | -1.9587738 | 0.918232 | 0.000125 | 0.002776 |
| **Clec2e** | 15.80342638 | -1.9517226 | 0.749979 | 4.31E-05 | 0.001244 |
| **Hoxd1** | 4.620696872 | -1.9486056 | 1.062435 | 0.000237 | 0.004408 |
| **Degs2** | 29.94688393 | -1.9385954 | 0.522389 | 2.56E-06 | 0.000156 |
| **Spink5** | 2570.451335 | -1.9373324 | 0.857064 | 9.36E-05 | 0.002257 |
| **Prom2** | 211.4537605 | -1.9302101 | 0.543004 | 3.87E-06 | 0.000211 |
| **Zcwpw1** | 60.56661496 | -1.9271398 | 0.830816 | 8.14E-05 | 0.002047 |
| **Sprr2b** | 13.40881607 | -1.9199535 | 1.523407 | 0.000618 | 0.008874 |
| **Mpp2** | 59.40673541 | -1.9113604 | 0.671065 | 2.31E-05 | 0.000804 |
| **H2-Q2** | 26.09855824 | -1.90989 | 0.841846 | 9.08E-05 | 0.002212 |
| **Cbs** | 24.93644932 | -1.9057807 | 0.505328 | 2.00E-06 | 0.000131 |
| **Atp13a5** | 7.875216253 | -1.8985691 | 0.89205 | 0.000125 | 0.002772 |
| **Rnf186** | 33.3900879 | -1.8909055 | 1.155553 | 0.000361 | 0.005978 |
| **Card14** | 73.3268397 | -1.8767352 | 0.545166 | 4.71E-06 | 0.000242 |
| **Erich6** | 7.585352621 | -1.8657324 | 1.35734 | 0.000583 | 0.008553 |
| **Dhrs9** | 632.1572113 | -1.8554831 | 0.770255 | 6.32E-05 | 0.001694 |
| **Myh10** | 453.054193 | -1.8397058 | 0.237878 | 2.49E-15 | 4.52E-12 |
| **Fgfbp1** | 882.2250324 | -1.8390101 | 0.632172 | 1.80E-05 | 0.000668 |
| **Xirp1** | 10.83047864 | -1.8375733 | 0.830037 | 9.97E-05 | 0.002366 |
| **Spdef** | 18.73389876 | -1.8359831 | 0.959695 | 0.000201 | 0.003931 |
| **Nuak1** | 1128.688701 | -1.8168271 | 0.279273 | 1.00E-11 | 5.81E-09 |
| **Gsta1** | 395.8962844 | -1.8091247 | 0.683591 | 3.37E-05 | 0.001037 |
| **Dpysl5** | 23.41614242 | -1.8040044 | 0.822825 | 0.000103 | 0.002431 |
| **Alox15** | 293.136037 | -1.7996339 | 1.123671 | 0.000403 | 0.006426 |
| **Slc4a5** | 12.63900449 | -1.7938481 | 0.873925 | 0.000144 | 0.003102 |
| **Cxcl10** | 676.4663447 | -1.7936777 | 0.531313 | 4.71E-06 | 0.000242 |
| **Tgm1** | 2701.997442 | -1.7837105 | 0.572554 | 9.49E-06 | 0.000415 |
| **Aldh3a1** | 381.8989354 | -1.7708776 | 1.030946 | 0.000314 | 0.005372 |
| **Syt9** | 13.26455025 | -1.7583063 | 1.058703 | 0.000358 | 0.005941 |
| **Cerkl** | 6.176858744 | -1.7327059 | 0.958235 | 0.000255 | 0.004645 |
| **Limch1** | 122.5572047 | -1.7175257 | 1.110737 | 0.000467 | 0.00717 |
| **Lypd5** | 113.4947446 | -1.7152106 | 1.475704 | 0.00089 | 0.011585 |
| **Fyb2** | 26.21621812 | -1.7128459 | 0.962573 | 0.000273 | 0.004854 |
| **Azgp1** | 9.115648742 | -1.7087402 | 1.732407 | 0.000868 | 0.011394 |
| **Cd5** | 37.68413251 | -1.6866749 | 0.57365 | 1.27E-05 | 0.000522 |
| **Scn4b** | 16.87666456 | -1.679939 | 1.100359 | 0.000495 | 0.007538 |
| **Rgs1** | 156.4002347 | -1.6554973 | 0.557717 | 1.10E-05 | 0.000473 |
| **Serpinb1b** | 16.84730095 | -1.6533467 | 0.722217 | 7.22E-05 | 0.001866 |
| **Fibin** | 190.4969104 | -1.6515374 | 0.927082 | 0.00027 | 0.004811 |
| **Lor** | 180.1919185 | -1.6503475 | 0.939611 | 0.000287 | 0.005058 |
| **Sprr2e** | 35.59167597 | -1.6465821 | 0.769537 | 0.000107 | 0.002497 |
| **Xist** | 13541.71311 | -1.6407671 | 1.363899 | 0.00094 | 0.012091 |
| **Bglap3** | 20.97085133 | -1.6328665 | 0.603595 | 2.24E-05 | 0.000788 |
| **Tmc3** | 29.98367361 | -1.6291065 | 1.047663 | 0.00047 | 0.007207 |
| **Atp6v0a4** | 17.84645959 | -1.6242786 | 0.833922 | 0.000175 | 0.003508 |
| **Apol6** | 95.37638458 | -1.6196595 | 0.626343 | 3.07E-05 | 0.000981 |
| **Megf6** | 533.2619428 | -1.6175151 | 1.120126 | 0.00061 | 0.008842 |
| **Tmem54** | 126.4072827 | -1.5894467 | 0.323652 | 8.92E-09 | 2.34E-06 |
| **Ccdc158** | 35.95197171 | -1.5725569 | 1.360077 | 0.001084 | 0.013397 |
| **Mlf1** | 11.63849191 | -1.556129 | 0.698999 | 7.83E-05 | 0.001991 |
| **Vstm5** | 21.55399457 | -1.5536617 | 0.681549 | 6.71E-05 | 0.001763 |
| **Dlg2** | 35.05097545 | -1.5505191 | 0.720346 | 9.58E-05 | 0.002304 |
| **Scube1** | 14.06966854 | -1.5320418 | 0.563794 | 1.80E-05 | 0.000668 |
| **Gbp9** | 564.8997897 | -1.5212512 | 0.552748 | 1.59E-05 | 0.00062 |
| **Car9** | 168.2554734 | -1.5171176 | 0.530039 | 1.12E-05 | 0.000479 |
| **Dmc1** | 5.16632568 | -1.5153717 | 1.554294 | 0.001312 | 0.015415 |
| **Slc28a3** | 113.9442678 | -1.5143612 | 1.278166 | 0.001108 | 0.013595 |
| **Ubd** | 171.3847047 | -1.5025669 | 0.932311 | 0.000414 | 0.006552 |
| **H2bc22** | 77.47320929 | -1.496791 | 0.480829 | 4.78E-06 | 0.000244 |
| **Klk14** | 295.9547709 | -1.4949467 | 1.242915 | 0.001094 | 0.013477 |
| **B3galt5** | 56.35039316 | -1.4831892 | 1.211329 | 0.00106 | 0.013205 |
| **Cst6** | 17.5428362 | -1.4785732 | 0.565047 | 2.23E-05 | 0.000785 |
| **Acer1** | 8.017216296 | -1.4344962 | 1.060691 | 0.00082 | 0.010954 |
| **Boc** | 133.3957158 | -1.4330839 | 0.365749 | 2.08E-07 | 2.44E-05 |
| **Sox11** | 914.6599643 | -1.4301008 | 1.0135 | 0.000708 | 0.009856 |
| **Slc1a3** | 37.24623956 | -1.4215469 | 0.800976 | 0.000261 | 0.00471 |
| **Rdh12** | 213.7341479 | -1.4072665 | 0.651679 | 8.26E-05 | 0.002069 |
| **Ndrg2** | 575.0578264 | -1.4046052 | 0.457944 | 4.31E-06 | 0.000228 |
| **Atf3** | 521.8145842 | -1.4040085 | 0.910323 | 0.000502 | 0.007588 |
| **Ptn** | 321.3033039 | -1.3830879 | 0.646462 | 8.60E-05 | 0.002131 |
| **Bambi** | 131.6295636 | -1.3821812 | 0.750045 | 0.000211 | 0.004057 |
| **Map2** | 441.1134912 | -1.3749539 | 0.503243 | 1.26E-05 | 0.000519 |
| **Abcg1** | 390.8611968 | -1.3570477 | 0.577568 | 4.32E-05 | 0.001244 |
| **Grb10** | 545.7458626 | -1.3534973 | 0.977026 | 0.000777 | 0.0106 |
| **Gnasas1** | 22.17283634 | -1.3321725 | 1.388672 | 0.001865 | 0.019734 |
| **Zfp286** | 10.33943548 | -1.3303413 | 1.182091 | 0.001481 | 0.01684 |
| **Tspoap1** | 57.0332587 | -1.3214283 | 0.472226 | 9.03E-06 | 0.000399 |
| **Ptprv** | 19.31952197 | -1.3077128 | 0.971109 | 0.000877 | 0.011468 |
| **Zdhhc15** | 104.4263113 | -1.3034667 | 0.791188 | 0.000371 | 0.006089 |
| **Lnx1** | 62.34953126 | -1.2973195 | 0.585664 | 6.27E-05 | 0.00169 |
| **Pi15** | 2029.421628 | -1.2963037 | 0.350024 | 2.64E-07 | 2.77E-05 |
| **Dnah8** | 133.2889966 | -1.2772717 | 0.831071 | 0.000515 | 0.007733 |
| **Shisa2** | 7.569316454 | -1.2742678 | 1.08904 | 0.001407 | 0.016185 |
| **Ly6g6g** | 41.2017971 | -1.2647555 | 0.441107 | 6.31E-06 | 0.000307 |
| **Afap1l2** | 310.0469695 | -1.2571762 | 0.303677 | 3.31E-08 | 6.32E-06 |
| **Espn** | 179.4843384 | -1.2445242 | 0.473019 | 1.43E-05 | 0.000571 |
| **Gramd1c** | 74.36182003 | -1.2442731 | 0.328519 | 1.51E-07 | 1.92E-05 |
| **Hpgd** | 81.7011759 | -1.2419082 | 0.872337 | 0.000722 | 0.010032 |
| **Rplp0-ps1** | 97.59640839 | -1.2396663 | 0.818896 | 0.000554 | 0.008233 |
| **Il36b** | 21.28823142 | -1.2256098 | 1.072281 | 0.00156 | 0.017405 |
| **Sntb1** | 59.51026904 | -1.2242656 | 0.826663 | 0.000613 | 0.008864 |
| **Ifi204** | 1550.697225 | -1.2237343 | 0.510862 | 3.21E-05 | 0.001008 |
| **Flrt1** | 132.8429564 | -1.2201606 | 0.518478 | 3.70E-05 | 0.001115 |
| **Lrrc8e** | 190.125107 | -1.2192942 | 0.404347 | 3.32E-06 | 0.000185 |
| **Car13** | 1147.998427 | -1.216189 | 0.644819 | 0.000175 | 0.003508 |
| **Srsf12** | 5.135520936 | -1.2091459 | 1.094969 | 0.001734 | 0.018799 |
| **Mb** | 10.67302299 | -1.2086342 | 0.848212 | 0.000727 | 0.010061 |
| **Wincr1** | 27.91849081 | -1.2085162 | 1.05072 | 0.001554 | 0.017351 |
| **Armc2** | 6.33408457 | -1.2054306 | 0.686812 | 0.000265 | 0.004741 |
| **Angptl6** | 12.23150114 | -1.1939484 | 0.534233 | 5.46E-05 | 0.001503 |
| **Msi1** | 48.71740614 | -1.1807139 | 1.090719 | 0.001873 | 0.019783 |
| **Xkrx** | 13.72762211 | -1.180222 | 0.858575 | 0.000852 | 0.011235 |
| **Syt14** | 15.89123307 | -1.1712478 | 1.219411 | 0.002406 | 0.023773 |
| **Cfap47** | 18.64399154 | -1.1694518 | 1.43749 | 0.002564 | 0.024873 |
| **Itgb3bp** | 41.40647216 | -1.1664514 | 0.191841 | 4.39E-13 | 3.75E-10 |
| **Mtus2** | 39.50752787 | -1.1646937 | 0.823413 | 0.000762 | 0.010438 |
| **Defb1** | 100.9851858 | -1.1594788 | 0.626414 | 0.000195 | 0.003825 |
| **Igtp** | 1196.657545 | -1.1572594 | 0.440966 | 1.35E-05 | 0.000546 |
| **Zfp808** | 50.86881331 | -1.1553028 | 0.339011 | 6.07E-07 | 5.10E-05 |
| **B4galnt3** | 128.0372972 | -1.1519565 | 0.399286 | 5.02E-06 | 0.000255 |
| **Ifng** | 7.852693322 | -1.1464035 | 0.736613 | 0.000498 | 0.007568 |
| **Sprr2i** | 34.12153241 | -1.141223 | 1.122105 | 0.002256 | 0.022702 |
| **Insl6** | 102.9763506 | -1.1266825 | 0.365017 | 2.22E-06 | 0.000141 |
| **Art3** | 144.5839411 | -1.1241047 | 0.843491 | 0.000992 | 0.01259 |
| **Capsl** | 153.3160725 | -1.1171064 | 0.896383 | 0.00128 | 0.015115 |
| **Otub2** | 269.262163 | -1.1163193 | 0.299743 | 1.44E-07 | 1.85E-05 |
| **Vsnl1** | 574.1732455 | -1.1145957 | 0.612732 | 0.000218 | 0.004144 |
| **Apoc1** | 12.90109648 | -1.1085484 | 1.20934 | 0.002818 | 0.026627 |
| **Gstm6** | 5.427537801 | -1.1001042 | 0.978541 | 0.001816 | 0.019453 |
| **Slco5a1** | 81.58948586 | -1.0977861 | 0.513731 | 7.57E-05 | 0.00194 |
| **Nell2** | 5.120242138 | -1.0880983 | 1.123466 | 0.002657 | 0.025449 |
| **Kcnk7** | 32.36324037 | -1.0825727 | 0.352794 | 2.37E-06 | 0.000146 |
| **Ces2b** | 18.25852189 | -1.0789696 | 1.26321 | 0.00317 | 0.028597 |
| **Selenbp1** | 169.8800829 | -1.0763736 | 0.380017 | 6.04E-06 | 0.000298 |
| **Hes2** | 36.7666549 | -1.0753698 | 1.466635 | 0.002898 | 0.027059 |
| **Tcam1** | 9.860605813 | -1.0705801 | 0.566725 | 0.000177 | 0.003538 |
| **Zfp92** | 136.0712923 | -1.0657837 | 0.904238 | 0.001611 | 0.017852 |
| **Rnf17** | 4.430545008 | -1.0524329 | 1.28878 | 0.003403 | 0.030142 |
| **Mdfi** | 267.8618733 | -1.051241 | 0.302298 | 4.32E-07 | 3.97E-05 |
| **Lce3a** | 61.82208712 | -1.0461731 | 1.134625 | 0.003072 | 0.028 |
| **Prf1** | 33.12921199 | -1.0424783 | 0.644066 | 0.000432 | 0.006765 |
| **Hebp2** | 545.7901057 | -1.0310013 | 0.579005 | 0.000259 | 0.004696 |
| **Hopx** | 1128.765156 | -1.0307741 | 0.855604 | 0.001541 | 0.017256 |
| **Sult2b1** | 460.6510946 | -1.0303947 | 0.761955 | 0.000996 | 0.012626 |
| **Kcnv1** | 30.28045884 | -1.0259802 | 1.062799 | 0.002899 | 0.027059 |
| **Pla2g3** | 12.38641935 | -1.0170452 | 0.907253 | 0.001978 | 0.020546 |
| **Dtna** | 49.19069645 | -1.0150153 | 1.170259 | 0.003519 | 0.03092 |
| **Gbp2** | 3050.725473 | -1.0069748 | 0.461031 | 6.92E-05 | 0.001805 |
| **Myzap** | 524.8406512 | -1.0008266 | 0.701387 | 0.000815 | 0.010926 |
| **Gdf10** | 14.48416066 | 1.0004657 | 1.179474 | 0.003699 | 0.03189 |
| **Flot2** | 473.8943183 | 1.0043685 | 0.607855 | 0.000402 | 0.006421 |
| **Hs3st3a1** | 35.11926405 | 1.005435 | 0.717828 | 0.000879 | 0.01147 |
| **Dpp6** | 16.12135703 | 1.0068771 | 1.122514 | 0.003408 | 0.030162 |
| **H2aj** | 595.024088 | 1.0147151 | 0.223502 | 2.86E-09 | 9.32E-07 |
| **Trhde** | 9.506202806 | 1.0154171 | 1.364027 | 0.003577 | 0.031155 |
| **Hao1** | 6.60144369 | 1.0181936 | 0.905724 | 0.001958 | 0.020393 |
| **Tm7sf3** | 328.8664744 | 1.0202547 | 0.131803 | 1.69E-18 | 4.90E-15 |
| **Hoxb5** | 338.910935 | 1.020443 | 0.587526 | 0.000302 | 0.005254 |
| **Pcdhga7** | 174.4696713 | 1.0259354 | 0.610563 | 0.00036 | 0.005959 |
| **Kcnn4** | 1463.475604 | 1.0284562 | 0.694351 | 0.000672 | 0.009507 |
| **Pcdhga4** | 108.8218996 | 1.0342285 | 0.461307 | 5.48E-05 | 0.001503 |
| **Fkrp** | 223.3170369 | 1.0344712 | 0.271369 | 9.81E-08 | 1.43E-05 |
| **Lacc1** | 312.2724564 | 1.0447111 | 0.339115 | 2.31E-06 | 0.000144 |
| **Arl4d** | 27.0363988 | 1.0496339 | 0.446392 | 3.68E-05 | 0.00111 |
| **Zfp398** | 775.1705233 | 1.0512246 | 0.244415 | 9.03E-09 | 2.34E-06 |
| **Polg** | 1249.082085 | 1.0527219 | 0.273777 | 8.21E-08 | 1.25E-05 |
| **Xlr3a** | 54.1150472 | 1.0532872 | 1.132808 | 0.002999 | 0.027616 |
| **Naip1** | 26.69334571 | 1.0567481 | 0.724385 | 0.000705 | 0.009819 |
| **Snora57** | 39.35200626 | 1.0583001 | 0.71505 | 0.00066 | 0.009365 |
| **Icam5** | 30.72670857 | 1.0603619 | 0.544807 | 0.000147 | 0.003144 |
| **Mmp7** | 10.18634349 | 1.0628542 | 1.577882 | 0.002415 | 0.023784 |
| **Fgf10** | 10.23859943 | 1.0674042 | 1.245224 | 0.003234 | 0.029068 |
| **B4galt5** | 725.0167432 | 1.071278 | 0.772299 | 0.00087 | 0.011399 |
| **Slc25a35** | 133.4435431 | 1.0715163 | 0.645802 | 0.000372 | 0.006109 |
| **Grin2d** | 55.91183535 | 1.0794487 | 0.595805 | 0.000227 | 0.004267 |
| **Ltbp4** | 4076.704787 | 1.0817215 | 0.389259 | 7.41E-06 | 0.000345 |
| **Pkn1** | 901.42941 | 1.0834874 | 0.275004 | 5.19E-08 | 8.81E-06 |
| **Pcdhb9** | 24.02714864 | 1.0924233 | 0.660511 | 0.000374 | 0.006137 |
| **Sptssb** | 61.93051134 | 1.0925397 | 1.065247 | 0.002338 | 0.023246 |
| **Igfbp5** | 1470.936114 | 1.0945603 | 0.980164 | 0.001862 | 0.019734 |
| **Dlk1** | 6.927391751 | 1.1019514 | 1.143642 | 0.002634 | 0.025303 |
| **Mgmt** | 58.67184181 | 1.1201566 | 0.970932 | 0.001651 | 0.018184 |
| **Klhl23** | 230.7110573 | 1.1222487 | 0.572624 | 0.000137 | 0.002976 |
| **Aqp3** | 4260.992042 | 1.1247123 | 0.739452 | 0.00056 | 0.008286 |
| **Clec2g** | 217.6212912 | 1.1284444 | 1.05295 | 0.002021 | 0.020892 |
| **Galnt4** | 791.8901085 | 1.1421155 | 0.321267 | 3.16E-07 | 3.11E-05 |
| **Lrch2** | 16.44992949 | 1.1470127 | 1.016411 | 0.001726 | 0.018732 |
| **Gldc** | 29.44282407 | 1.1493195 | 0.874326 | 0.001031 | 0.01298 |
| **Tmem238** | 170.8305534 | 1.1495566 | 0.312972 | 1.90E-07 | 2.26E-05 |
| **Dnase1l1** | 169.6848854 | 1.1530337 | 0.463813 | 2.22E-05 | 0.000785 |
| **Ercc1** | 400.5770761 | 1.1567707 | 0.61172 | 0.000171 | 0.003461 |
| **Cd55** | 502.8746031 | 1.1602408 | 1.049382 | 0.001806 | 0.019379 |
| **Mgat5b** | 57.93147604 | 1.1636643 | 1.10979 | 0.002057 | 0.021156 |
| **Rgs9bp** | 13.42594164 | 1.1682929 | 0.71043 | 0.000376 | 0.00614 |
| **Gpd1** | 19.72342957 | 1.1705875 | 1.090039 | 0.001929 | 0.020174 |
| **Orai2** | 729.0116078 | 1.1735796 | 0.626982 | 0.000182 | 0.003625 |
| **Suox** | 211.3540137 | 1.1906314 | 0.595841 | 0.000121 | 0.002716 |
| **Txnip** | 1901.374822 | 1.1941325 | 0.431265 | 8.14E-06 | 0.000372 |
| **Sox5os3** | 8.086135575 | 1.2010076 | 1.307374 | 0.002412 | 0.023773 |
| **Ptgs2os2** | 49.40268693 | 1.203629 | 0.812807 | 0.000617 | 0.008874 |
| **Klhl29** | 167.3361804 | 1.2050124 | 0.786476 | 0.000528 | 0.007918 |
| **Bcl2l15** | 161.5629702 | 1.2064944 | 1.079705 | 0.001686 | 0.018395 |
| **Zfp784** | 36.0436579 | 1.2115897 | 0.464619 | 1.50E-05 | 0.000595 |
| **Pcdh11x** | 7.167135436 | 1.2262849 | 1.487093 | 0.002242 | 0.022616 |
| **Nos1ap** | 53.37868495 | 1.2267031 | 0.649963 | 0.000174 | 0.003507 |
| **Hdac9** | 119.8357886 | 1.2382231 | 0.799773 | 0.0005 | 0.007577 |
| **Ccdc9b** | 799.6166298 | 1.240746 | 0.330148 | 1.69E-07 | 2.08E-05 |
| **Ang** | 72.34524181 | 1.2417532 | 0.415703 | 3.86E-06 | 0.000211 |
| **Zfpm1** | 244.9395035 | 1.2465041 | 0.42083 | 4.28E-06 | 0.000228 |
| **Man2a2** | 1868.797512 | 1.2495762 | 0.243888 | 2.35E-10 | 9.79E-08 |
| **Tmem191c** | 17.2983713 | 1.2603051 | 0.51058 | 2.59E-05 | 0.000868 |
| **Qpctl** | 225.3364547 | 1.2718969 | 0.364253 | 5.76E-07 | 4.98E-05 |
| **Grm7** | 4.612052231 | 1.2727119 | 1.675078 | 0.001654 | 0.018185 |
| **Sarm1** | 12.34239603 | 1.2746145 | 0.828337 | 0.000512 | 0.007725 |
| **Ceacam12** | 34.95501284 | 1.2777451 | 0.703889 | 0.000221 | 0.004189 |
| **Areg** | 819.8669204 | 1.304559 | 0.943641 | 0.000796 | 0.010788 |
| **Rnase4** | 565.381932 | 1.3215085 | 0.423829 | 2.85E-06 | 0.000166 |
| **Cyp2a5** | 3.934708885 | 1.3219534 | 1.531984 | 0.001848 | 0.019641 |
| **Syna** | 15.04007418 | 1.3273632 | 1.109417 | 0.001271 | 0.015054 |
| **Tmprss11a** | 1115.068893 | 1.3320339 | 1.173247 | 0.001448 | 0.016552 |
| **Slc41a2** | 153.2745818 | 1.3372623 | 0.766517 | 0.000279 | 0.004931 |
| **Cpe** | 2479.667658 | 1.3522579 | 0.619746 | 7.33E-05 | 0.00189 |
| **Stxbp6** | 104.4385495 | 1.3570395 | 1.200664 | 0.001429 | 0.016411 |
| **Kcnc2** | 19.82986196 | 1.3831953 | 1.15952 | 0.001227 | 0.014671 |
| **Nova2** | 92.1056778 | 1.3894151 | 0.468134 | 5.83E-06 | 0.000288 |
| **Nova1** | 45.01676823 | 1.396682 | 1.162726 | 0.001193 | 0.014436 |
| **Mfge8** | 2857.607932 | 1.3976974 | 0.249708 | 6.74E-11 | 3.37E-08 |
| **Ccdc142** | 34.92181099 | 1.3993544 | 0.456116 | 4.23E-06 | 0.000226 |
| **Cadps** | 40.08534601 | 1.4058668 | 1.431315 | 0.00162 | 0.017926 |
| **Rasgrf2** | 29.03872782 | 1.4167964 | 1.11525 | 0.001007 | 0.012743 |
| **Map6** | 455.2758511 | 1.4195786 | 0.8891 | 0.000431 | 0.006765 |
| **Ccdc92b** | 8.758626748 | 1.4288318 | 0.805915 | 0.000263 | 0.004727 |
| **Zfp462** | 765.2898357 | 1.4316311 | 0.793569 | 0.00024 | 0.004443 |
| **Lrrc8b** | 862.0610212 | 1.44045 | 0.225779 | 1.19E-12 | 8.62E-10 |
| **Pknox2** | 27.05413995 | 1.4412983 | 0.954702 | 0.000546 | 0.008146 |
| **Arl10** | 73.89604019 | 1.4608368 | 0.33643 | 4.38E-08 | 7.79E-06 |
| **Tmcc2** | 130.1370503 | 1.4742642 | 0.532695 | 1.38E-05 | 0.000553 |
| **Rnf169** | 1616.774153 | 1.4777086 | 0.329954 | 2.74E-08 | 5.61E-06 |
| **Mme** | 19.5833439 | 1.4876289 | 1.291374 | 0.001203 | 0.014499 |
| **Slitrk1** | 24.30149104 | 1.4909521 | 1.308387 | 0.001222 | 0.014631 |
| **Hcn4** | 9.120135855 | 1.505434 | 1.059884 | 0.000674 | 0.009528 |
| **Il11** | 15.02835679 | 1.5569741 | 0.901086 | 0.000306 | 0.005306 |
| **Atp2a1** | 10.51242649 | 1.5570248 | 1.18709 | 0.00084 | 0.011144 |
| **Fa2h** | 8.490010473 | 1.5605264 | 1.290098 | 0.001015 | 0.012821 |
| **H2bc21** | 41.21379053 | 1.5820595 | 0.4509 | 1.71E-06 | 0.000115 |
| **Scrn1** | 22.30969576 | 1.5892064 | 1.067149 | 0.000556 | 0.008236 |
| **Scn9a** | 7.374000962 | 1.5950913 | 1.97537 | 0.000751 | 0.010315 |
| **Tmem74** | 112.2582456 | 1.6002794 | 1.216246 | 0.000808 | 0.010888 |
| **Igdcc4** | 51.7597516 | 1.6165151 | 0.876845 | 0.000229 | 0.004288 |
| **Otud7a** | 12.80940728 | 1.6395059 | 1.431606 | 0.001006 | 0.012737 |
| **Col27a1** | 152.4146027 | 1.6457574 | 0.896043 | 0.000235 | 0.004362 |
| **Tnfrsf22** | 312.9916225 | 1.6535405 | 0.351164 | 3.05E-08 | 5.94E-06 |
| **Bean1** | 17.47196095 | 1.6578224 | 1.187533 | 0.000662 | 0.009388 |
| **Cyp2u1** | 43.08128537 | 1.6660727 | 0.582965 | 1.54E-05 | 0.000611 |
| **Hal** | 19.13598603 | 1.673205 | 1.067307 | 0.000452 | 0.006993 |
| **Cryab** | 137.4600986 | 1.6866269 | 1.0868 | 0.000466 | 0.007167 |
| **Enox1** | 97.81894144 | 1.7051618 | 0.552033 | 8.69E-06 | 0.000387 |
| **Scara5** | 66.99381884 | 1.7075792 | 1.229807 | 0.00065 | 0.009279 |
| **Notum** | 31.11111565 | 1.712708 | 1.183902 | 0.000578 | 0.008508 |
| **Fam83e** | 101.2382086 | 1.7368772 | 0.483104 | 2.14E-06 | 0.000137 |
| **Htr1b** | 579.8425145 | 1.752472 | 1.160534 | 0.000497 | 0.007567 |
| **Golm1** | 423.9283615 | 1.7534364 | 0.424487 | 4.08E-07 | 3.80E-05 |
| **Lrp3** | 23.72019993 | 1.7804725 | 0.882133 | 0.000155 | 0.00326 |
| **Amdhd1** | 24.42094004 | 1.7840165 | 0.879401 | 0.000152 | 0.003223 |
| **Itm2a** | 87.6165537 | 1.7895247 | 0.991782 | 0.000258 | 0.004677 |
| **Nlrp5-ps** | 17.67872313 | 1.8032477 | 1.206659 | 0.000499 | 0.007568 |
| **Cfap97d2** | 31.10291962 | 1.8053774 | 1.26361 | 0.000563 | 0.0083 |
| **Dsc3** | 3242.392948 | 1.8091315 | 0.391924 | 9.13E-08 | 1.37E-05 |
| **Ggct** | 1377.763124 | 1.8345538 | 1.104257 | 0.000351 | 0.005856 |
| **Sox5** | 441.1458403 | 1.8458988 | 0.997957 | 0.000232 | 0.004334 |
| **Npy4r** | 68.44886149 | 1.8464253 | 0.411134 | 1.66E-07 | 2.06E-05 |
| **Tchh** | 2731.424479 | 1.8538755 | 0.500916 | 2.15E-06 | 0.000137 |
| **Igfbp4** | 2100.634862 | 1.8611375 | 0.690259 | 3.16E-05 | 0.001 |
| **Calca** | 120.9271622 | 1.871623 | 0.995217 | 0.000217 | 0.004134 |
| **Vegfc** | 66.84516458 | 1.889851 | 0.847657 | 9.85E-05 | 0.002352 |
| **Serpina3j** | 5.215904239 | 1.9007699 | 1.730936 | 0.000644 | 0.009222 |
| **Slc16a11** | 724.0338453 | 1.9068357 | 0.853542 | 9.81E-05 | 0.002348 |
| **En1** | 22.51228104 | 1.9206862 | 0.895152 | 0.000121 | 0.002716 |
| **Celf4** | 390.0167611 | 1.9359833 | 0.467208 | 7.03E-07 | 5.75E-05 |
| **Rasa4** | 485.8681639 | 1.9374126 | 0.764508 | 5.00E-05 | 0.001402 |
| **Zfp449** | 30.2790479 | 1.9574918 | 0.41114 | 8.97E-08 | 1.36E-05 |
| **Dbp** | 398.9532959 | 1.9740013 | 0.670927 | 1.98E-05 | 0.000719 |
| **Fbxw9** | 395.3052423 | 1.9859463 | 0.441134 | 2.48E-07 | 2.71E-05 |
| **Prdm8** | 22.65371544 | 2.0026858 | 1.296484 | 0.000398 | 0.00639 |
| **Ltb4r2** | 385.429902 | 2.0084819 | 0.451017 | 3.11E-07 | 3.11E-05 |
| **Rasgef1a** | 25.29499604 | 2.0371301 | 1.272738 | 0.000354 | 0.005892 |
| **Ltb4r1** | 142.1901983 | 2.0439425 | 0.486206 | 7.58E-07 | 6.01E-05 |
| **Cnnm1** | 10.35189774 | 2.060027 | 1.724742 | 0.000498 | 0.007567 |
| **Msx3** | 40.14843644 | 2.0666219 | 0.678479 | 1.71E-05 | 0.000647 |
| **Msx1** | 57.41697593 | 2.0728922 | 0.559635 | 3.36E-06 | 0.000187 |
| **Slc4a8** | 171.9454007 | 2.1331545 | 1.011928 | 0.000133 | 0.002912 |
| **Tex14** | 34.23564727 | 2.1823047 | 1.271362 | 0.000264 | 0.004737 |
| **Vmn1r219** | 8.078096171 | 2.1880483 | 2.160671 | 0.000317 | 0.005404 |
| **Pcdhb7** | 19.40179205 | 2.2155477 | 0.683934 | 1.25E-05 | 0.000517 |
| **Tspan8** | 263.2749677 | 2.2168996 | 0.789566 | 3.22E-05 | 0.001008 |
| **Pcdhb5** | 22.13589683 | 2.2218756 | 0.507035 | 6.00E-07 | 5.10E-05 |
| **Scg2** | 10.5376824 | 2.2345342 | 1.933092 | 0.000348 | 0.005827 |
| **Igfl3** | 11.78606902 | 2.2484909 | 1.035718 | 0.000115 | 0.00263 |
| **Ntm** | 12.4469371 | 2.2512439 | 1.589252 | 0.000348 | 0.005827 |
| **Pcdhb8** | 7.793123815 | 2.2630246 | 0.994392 | 9.42E-05 | 0.002269 |
| **Sytl2** | 255.4553131 | 2.270826 | 0.687868 | 1.13E-05 | 0.000483 |
| **Hoxb8** | 226.2785141 | 2.2717539 | 0.739183 | 1.88E-05 | 0.000691 |
| **Pate14** | 4.349726137 | 2.2767279 | 1.531813 | 0.00032 | 0.005441 |
| **Pigr** | 92.05696775 | 2.2893919 | 0.963594 | 7.76E-05 | 0.001982 |
| **Abhd2** | 6780.272797 | 2.3053948 | 0.48708 | 2.02E-07 | 2.39E-05 |
| **Bmp3** | 229.6307465 | 2.3478033 | 0.74775 | 1.69E-05 | 0.000642 |
| **Cck** | 8.535990098 | 2.371105 | 1.844678 | 0.000292 | 0.005108 |
| **Synpr** | 11.43153035 | 2.3870949 | 1.512482 | 0.000256 | 0.004658 |
| **Dio2** | 262.7410088 | 2.4237358 | 1.132841 | 0.000114 | 0.002608 |
| **Astn2** | 11.29403568 | 2.4304446 | 1.077086 | 9.30E-05 | 0.002249 |
| **Atp6v0e2** | 30.47102159 | 2.4754309 | 1.285021 | 0.000153 | 0.003239 |
| **Slc44a4** | 67.15459784 | 2.4978103 | 0.64098 | 3.15E-06 | 0.000179 |
| **Pnp2** | 40.96982034 | 2.5270751 | 1.067818 | 7.39E-05 | 0.0019 |
| **Sema3a** | 286.0951627 | 2.531626 | 1.026104 | 6.21E-05 | 0.001676 |
| **Trim7** | 415.8201331 | 2.5377511 | 0.311628 | 1.36E-16 | 3.29E-13 |
| **Cpt1c** | 195.1508452 | 2.5509719 | 0.854373 | 2.39E-05 | 0.000819 |
| **Ptprn** | 223.7117717 | 2.5881416 | 1.005603 | 5.05E-05 | 0.001409 |
| **U90926** | 33.30044041 | 2.6146026 | 1.456077 | 0.000157 | 0.003273 |
| **Fras1** | 328.5814205 | 2.6251566 | 0.819777 | 1.56E-05 | 0.000614 |
| **Il13ra2** | 52.74121121 | 2.6400706 | 0.797277 | 1.25E-05 | 0.000517 |
| **Aqp9** | 83.10426396 | 2.6555324 | 0.560305 | 2.45E-07 | 2.70E-05 |
| **Pcdhb11** | 26.06061786 | 2.7453715 | 0.621246 | 7.51E-07 | 6.00E-05 |
| **Ndn** | 45.31584633 | 2.7530602 | 0.952604 | 2.72E-05 | 0.000903 |
| **Rgs17** | 171.184948 | 2.7567805 | 0.682474 | 2.29E-06 | 0.000143 |
| **Gpr27** | 98.61940112 | 2.8302489 | 0.991589 | 2.78E-05 | 0.000917 |
| **Tmem132b** | 13.39504982 | 2.8477351 | 1.153129 | 5.12E-05 | 0.001423 |
| **Anxa10** | 10.8929296 | 2.8503272 | 1.19567 | 5.79E-05 | 0.001569 |
| **Adamts15** | 521.673434 | 2.9296403 | 0.412415 | 2.93E-13 | 3.03E-10 |
| **Ube2ql1** | 24.71375258 | 2.9409022 | 0.728828 | 2.23E-06 | 0.000141 |
| **Cd55os** | 46.90010612 | 2.9745594 | 1.036178 | 2.47E-05 | 0.000837 |
| **Arhgef38** | 64.88222264 | 3.080457 | 1.313697 | 4.84E-05 | 0.001379 |
| **Stfa3** | 112.2972871 | 3.131081 | 0.618138 | 5.07E-08 | 8.77E-06 |
| **Olfr857** | 10.178103 | 3.1549756 | 0.934019 | 8.72E-06 | 0.000388 |
| **Clca1** | 52.46165882 | 3.1631008 | 0.817607 | 3.00E-06 | 0.000173 |
| **Col6a5** | 55.88110813 | 3.2257896 | 1.338672 | 3.76E-05 | 0.001124 |
| **Pcdhb10** | 10.47283523 | 3.2453679 | 0.664258 | 9.97E-08 | 1.43E-05 |
| **Calr3** | 76.24809571 | 3.5082743 | 0.847648 | 1.04E-06 | 7.76E-05 |
| **Gja3** | 31.42091948 | 3.6644879 | 1.337552 | 1.39E-05 | 0.000557 |
| **Necab2** | 38.44307339 | 3.7554166 | 1.415429 | 1.38E-05 | 0.000553 |
| **Chil4** | 540.9323987 | 3.788207 | 3.520994 | 3.92E-05 | 0.001156 |
| **Chrm3** | 33.80092767 | 3.7973399 | 1.244172 | 7.26E-06 | 0.00034 |
| **Add2** | 736.7125247 | 3.7978032 | 1.188195 | 5.71E-06 | 0.000283 |
| **Mtcl1** | 134.694718 | 3.8236327 | 0.734164 | 8.49E-09 | 2.28E-06 |
| **Tnfrsf19** | 122.9090633 | 3.8672819 | 0.763352 | 1.54E-08 | 3.40E-06 |
| **Foxp2** | 29.87964421 | 3.8838844 | 0.901894 | 3.35E-07 | 3.27E-05 |
| **Il5ra** | 118.6120699 | 4.0121342 | 2.195403 | 2.38E-05 | 0.000818 |
| **Olfr1189** | 4.069649111 | 4.0176999 | 1.052505 | 1.09E-06 | 8.06E-05 |
| **Plk5** | 22.10797999 | 4.0776356 | 1.110967 | 1.40E-06 | 9.90E-05 |
| **Egr4** | 28.47276924 | 4.1187171 | 0.997925 | 3.69E-07 | 3.52E-05 |
| **Rims2** | 50.66341491 | 4.17551 | 1.169004 | 1.47E-06 | 0.000103 |
| **Adgrg7** | 31.11005245 | 4.2607121 | 1.432005 | 4.12E-06 | 0.000222 |
| **Ntrk2** | 152.4478432 | 4.2756849 | 0.82856 | 3.36E-09 | 1.04E-06 |
| **Ppp1r14a** | 178.0040286 | 4.3942266 | 0.889377 | 6.92E-09 | 1.90E-06 |
| **Olfr858-ps1** | 11.69553185 | 4.4274854 | 0.922315 | 1.21E-08 | 2.87E-06 |
| **Gspt2** | 30.92830518 | 4.4646402 | 0.774858 | 7.25E-11 | 3.51E-08 |
| **Krt90** | 1906.927761 | 4.7436437 | 1.25818 | 2.80E-07 | 2.90E-05 |
| **Tafa5** | 22.93367285 | 4.7568562 | 1.086124 | 2.92E-08 | 5.81E-06 |
| **Magel2** | 37.63977297 | 5.1250324 | 2.776116 | 8.57E-06 | 0.000383 |
| **Foxl2** | 44.90209035 | 5.2671241 | 2.685463 | 6.68E-06 | 0.000323 |
| **Foxl2os** | 27.18164895 | 5.4606357 | 1.592474 | 2.38E-07 | 2.66E-05 |
| **Kdm5d** | 145.9858374 | 8.9584133 | 1.069696 | 3.05E-19 | 1.80E-15 |
| **Uty** | 129.3094503 | 9.276041 | 1.12627 | 1.59E-18 | 4.90E-15 |
| **Eif2s3y** | 127.6322016 | 9.7128678 | 1.30829 | 1.20E-15 | 2.50E-12 |
| **Ddx3y** | 132.631724 | 10.014981 | 1.042507 | 2.40E-23 | 3.49E-19 |
